# Immune persistence and response to booster dose of Vi-DT vaccine at 27.5 months post-first dose

**DOI:** 10.1038/s41541-022-00434-8

**Published:** 2022-01-27

**Authors:** Maria Rosario Capeding, Birkneh Tilahun Tadesse, Arijit Sil, Edison Alberto, Deok Ryun Kim, Eun Lyeong Park, Ju Yeon Park, Jae Seung Yang, Jagadeesh Reddy Eluru, Sue-Kyoung Jo, Hun Kim, Seon-Young Yang, Ji Hwa Ryu, Hokeun Park, Jong Hoon Shin, Yoonyeong Lee, Jerome H. Kim, Zenaida Reynoso Mojares, T. Anh Wartel, Sushant Sahastrabuddhe

**Affiliations:** 1 Research Institute for Tropical Medicine, Manila, The Philippines; 2 International Vaccine Institute, Seoul, Republic of Korea; 3 SK bioscience, Seoul, Republic of Korea

**Keywords:** Bacterial infection, Conjugate vaccines

## Abstract

Vaccination with typhoid conjugate vaccines (TCV) is a major part of typhoid prevention. However, little is known about long-term immune persistence following vaccination with TCVs. In this phase-2, randomized double-blind trial (NCT03527355), 285 children aged 6–23 months were randomized to one of three groups: (1) the group that received a first dose of Vi polysaccharide conjugated to diphtheria-toxoid (Vi-DT) vaccine followed by an “early booster” at 24 weeks, (2) the group that which received a first dose of Vi-DT followed by a “late booster” at 96 or 110 weeks, and (3) comparator group. Safety and immunogenicity of anti-Vi IgG GMTs were assessed at weeks 0, 4, 24, 28, 60, 96, 110, and 114 since the first dose. Here, we describe persistence of immune responses at weeks 60, 96, 110, and 114 post first dose. The anti-Vi IgG seroconversion rate after 27.5 months of follow-up was 88.16% (95% CI: 79.00, 93.64) in late-booster and 94.76% (95% CI: 86.91, 97.88) in early booster Vi-DT groups (*p* = 0.081). Whereas anti-Vi IgG GMTs were significantly higher in the early booster group (11.95 [95% CI: 9.65, 14.81]) than prebooster GMTs in the late booster group (5.50 [95% CI: 4.44, 6.80], *p* < 0.0001). GMT in the late booster group significantly increased to 351.76 (95% CI: 265.01, 466.93) (*p* < 0.0001) when measured 4 weeks after they received their “late-booster” shot. In conclusion, late booster dosing with Vi-DT at 27.5 months post first dose was safe and elicited robust anti-Vi IgG immune responses. Anti-Vi IgG seroconversion rates were persistently comparable in early and late-booster Vi-DT groups.

## Introduction

Typhoid is a significant public health problem in many developing countries where water sanitation and hygiene (WASH) behaviors and facilities require substantial modernization^1,2^. Globally, based on the Global Burden of Diseases estimate, typhoid fever affects an average of 15 million people annually with an all-age case-fatality rate of 0.95% (95% confidence interval (95% CI): 0.54–1.53)^2^. Infants and children are particularly at higher risk of mortality and morbidity from complications of typhoid fever^3^. In 2017, 55.9% (95% CI: 50.3–61.6) of typhoid episodes occurred in children younger than 15 years of age with an estimated case fatality rate of 1.6% (95% CI: 0.8–3.0), which is much higher than that observed in adults^2^. This problem is further compounded by recent reports of multidrug resistance and extremely drug-resistant *Salmonella enterica* serovar Typhi (*S*. Typhi) strains in several countries^4–6^. Safe and effective typhoid vaccines in conjunction with modest improvements in WASH facilities and behaviors present a unique opportunity to control and potentially eliminate typhoid in high-burden settings.

Polysaccharide vaccines are not immunogenic in children younger than 2 years of age due to the “thymus-independent” immune responses to these antigens in this age group, leading to limited development and maturation of memory B cells, and antibody-affinity maturation of the responses^7,8^. The unfavorable performance of *S*. Typhi polysaccharide vaccines in young children necessitated the development of typhoid-conjugate vaccines (TCVs). Over the past decade, there has been significant progress in the development of TCVs, which were shown to be safe, well-tolerated, immunogenic, and protective against typhoid fever across age groups^9–13^.

One of the typhoid-conjugate vaccines, Vi-DT (Vi polysaccharide conjugated with diphtheria toxoid) has been developed using conjugation of the Vi-polysaccharide antigen with diphtheria toxoid. In a previous analysis, we showed that Vi-DT is well-tolerated, shows a good safety profile, and can elicit robust immune responses at 6 months following the first dose of vaccination^14–17^. An important aspect of the typhoid-conjugate vaccine research includes the immune persistence following the period after vaccination, the need for and timing of a booster dose, and immune responses following a booster dose. For example, in the case of the tetanus toxoid-conjugated typhoid vaccine (Vi-TT, Typbar-TCV^®^), booster dosing at 2 years after the first dose demonstrated robust immune responses^18^. It is however important to note that there is limited understanding of the long-term persistence of immune responses, and the protection against typhoid fever, following vaccination using a single dose of approved and new-generation typhoid-conjugate vaccines.

In this analysis, we investigated two important aspects of typhoid vaccination using Vi-DT. First, we evaluated the immune persistence at 27.5 months in two Vi-DT groups—the first group receiving an early booster at 24 weeks after the first dose, the “early booster” group, and the second group receiving a late booster at 96 or 110 weeks after the first dose, the “late booster” group. Second, we assessed the safety and immune response at 4 weeks following a booster dose of Vi-DT administered at 27.5 months of the first dose in the late-booster group.

## Results

**Fig. 1:**
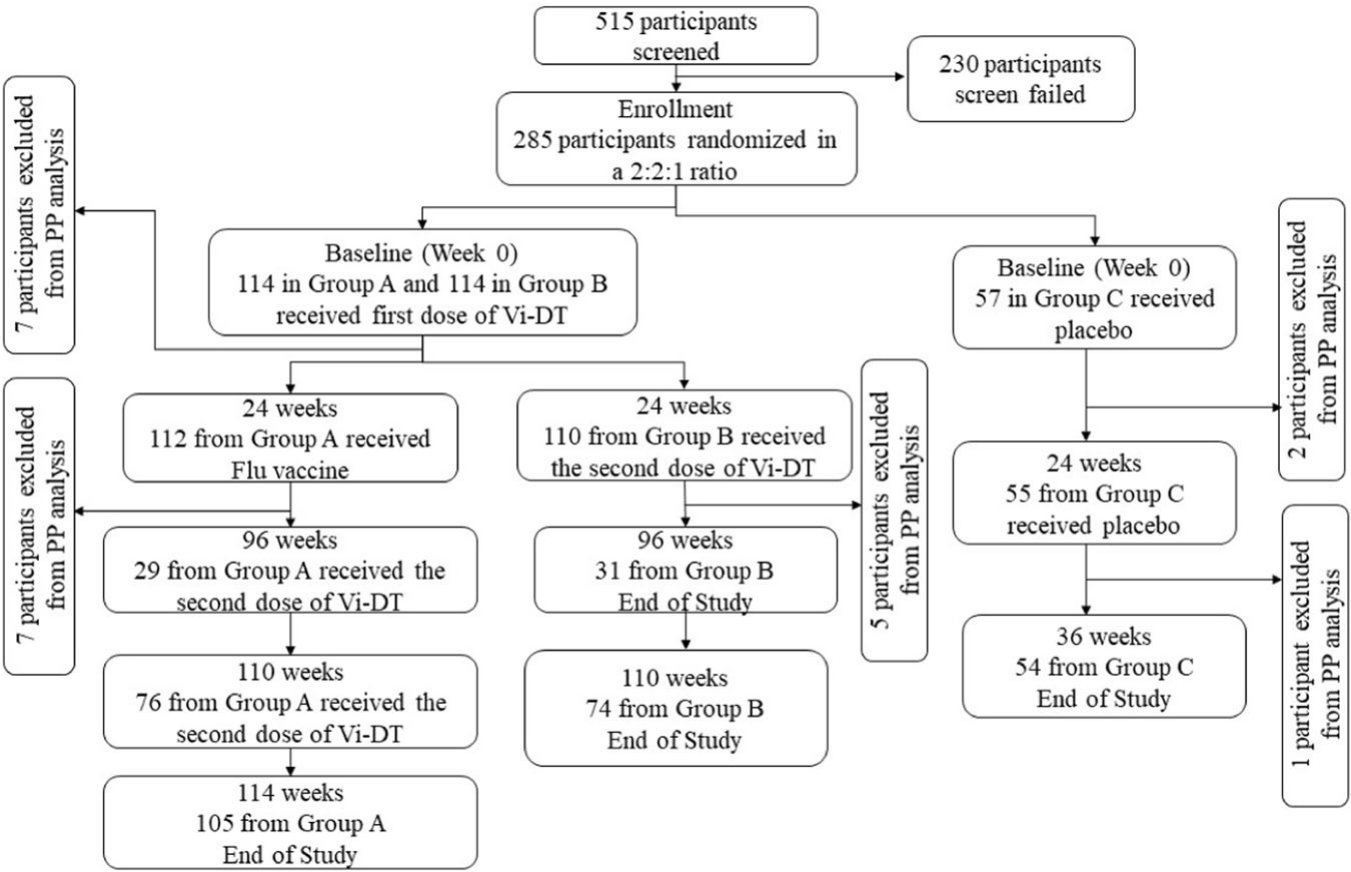
Flow diagram of participant disposition (CONSORT flow diagram). PP—per-protocol analysis. Group A represents the late-booster group, Group B represents the early booster group, and Group C represents the comparator group. A total of 230 potential participants failed screening at enrollment for not fulfilling the inclusion/exclusion criteria. The most common reasons for screen failure included abnormal screening laboratory values, including deranged liver enzymes, anemia, and abnormal hematology—77 (33.5%); withdrew consent at enrollment—48 (20.9%), had acute illnesses as a reason for presentation to the facility—45 (19.6%); refused consent at screening—25 (10.9%); parents/legal authorized guardians indicated that they have a plan to move out of the study area and will not be able to follow study procedures—22 (9.6%). Seven participants (3%) reported allergy to eggs, chicken protein, neomycin, and formaldehyde; 4 (1.7%) had previously ascertained or suspected disease caused by S. Typhi; and 2 (0.9%) had a congenital anomaly.

A total of 285 children were enrolled from 515 screened participants. The reasons for screen failure are presented in Fig. 1. All participants randomized at baseline were included in the immunogenicity and safety-analysis sets. In the per-protocol analysis, 109 (96%), 105 (92%), and 54 (95%) participants from the late-booster, early booster, and comparator groups were included, respectively. Two participants did not receive the second dose of Vi-DT, while five participants—two each in the late booster and early booster groups, and one participant in the comparator group—had delayed 6-month vaccination. An additional participant each from the late-booster and early booster Vi-DT groups, was excluded at the time point when each received their booster shot, due to the administrators missing their blood sample to conduct an immunogenicity test. Disposition of study participants during the study period is presented in Fig. 1.

### Persistence of seroconversion and GMTs at 110-week follow-up

We evaluated the persistence of immune responses at week 110 to the Vi-DT vaccine in the early booster group and later booster group prior to booster dosing using the immunogenicity-analysis set. We observed that the anti-Vi IgG seroconversion rates remained high at week 110 since the first vaccination. There was no significant difference between the prebooster seroconversion rates in the late-booster group and the post-booster rates in the early booster group at weeks 60, 96, and 110 post first vaccination. Seroconversion rate at week 60 was 95.50% (95% CI: 89.89, 98.06) and 96.33% (95% CI: 90.94, 98.56) in the late-booster and early booster groups, respectively (*p* = 0.757). Persistently high seroconversion rates were observed at weeks 96 and 110: seroconversion rate of 89.66% (95% CI: 73.61, 96.42) in the late-booster group and 96.77% (95% CI: 83.81, 99.43) in the early booster group at week 96 (*p* = 0.304); and, 88.16% (95% CI: 79.00, 93.64) in the late-booster group and 94.59% (95% CI: 86.91, 97.88) in the early booster Vi-DT group at week 110 (*p* = 0.162).

**Fig. 2:**
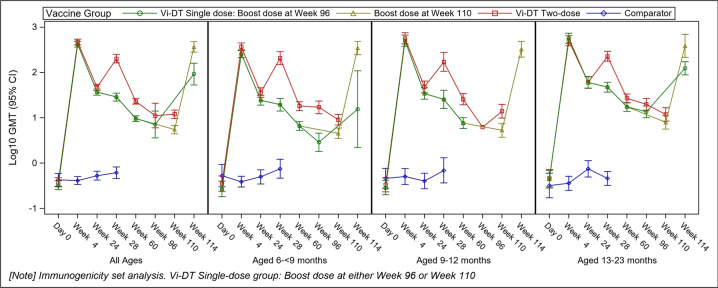
Log-transformed titer of anti-Vi IgG ELISA response using the immunogenicity analysis set. The *x* axis represents the immunogenicity blood-draw timepoints including for all age groups and by age group, while the *y* axis presents the log_10_-transformed geometric mean titers (GMTs). The dotted vertical lines represent the vaccination timepoints.

**Fig. 3:**
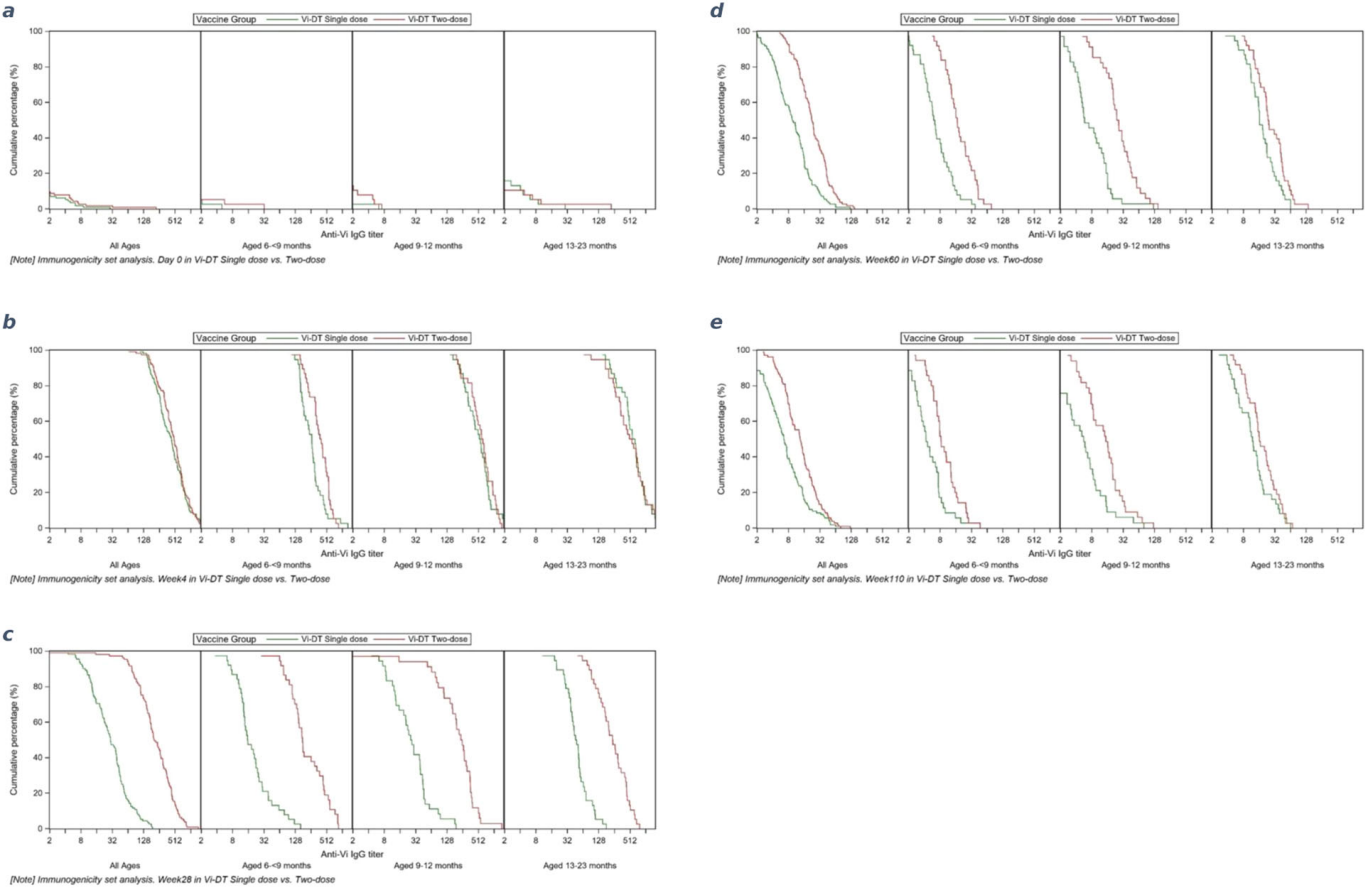
**a**–**e**: Reverse cumulative distribution curves (RCDC) showing anti-Vi IgG titers at predefined timepoints. **a** RCDC of the geometric mean titers in the three treatment groups at baseline. **b** RCDC of the geometric mean titers in the three treatment groups at 4 weeks. **c** RCDC of the geometric mean titers in the three treatment groups at 28 weeks. **d** RCDC of the geometric mean titers in the three treatment groups at 60 weeks. **e** RCDC of the geometric mean titers in the three treatment groups at 110 weeks. The *y* axis in each RCDC represents the cumulative percentage of participants who had the measured corresponding geometric mean titer (GMTs) (IU/L) displayed on the *x* axis for each visit. The *x* axis represents the GMTs measured for the respective visit for all ages and disaggregated by age.

Anti-Vi IgG GMTs were however proportionately higher in the early booster group than the prebooster values in the late booster Vi-DT group at each follow-up visit. At week 60, the anti-Vi IgG GMT was 9.60 (95% CI: 8.34, 11.05) and 23.01 (95% CI: 19.96, 26.52) in the late-booster and early booster groups, respectively (*p* < 0.0001). The GMTs decreased to 7.13 (95% CI: 3.60, 14.14) in the late-booster group and 11.18 (95% CI: 5.97, 20.90) in the early booster group at week 96 (*p* = 0.034), and to 5.50 (95% CI: 4.44, 6.80) in the late-booster group and 11.95 (95% CI: 9.65, 14.81) in the early booster group at week 110 (*p* < 0.0001). These GMTs at weeks 96 and 110 represent the prebooster values in the late-booster group. The differences in GMTs between prebooster values in the late-booster and those in the early booster groups by age strata at different timepoints are presented in Fig. 2, and the distribution of the GMTs between the groups at each immunogenicity blood-draw timepoint is presented in Fig. 3A–E.

**Fig. 4:**
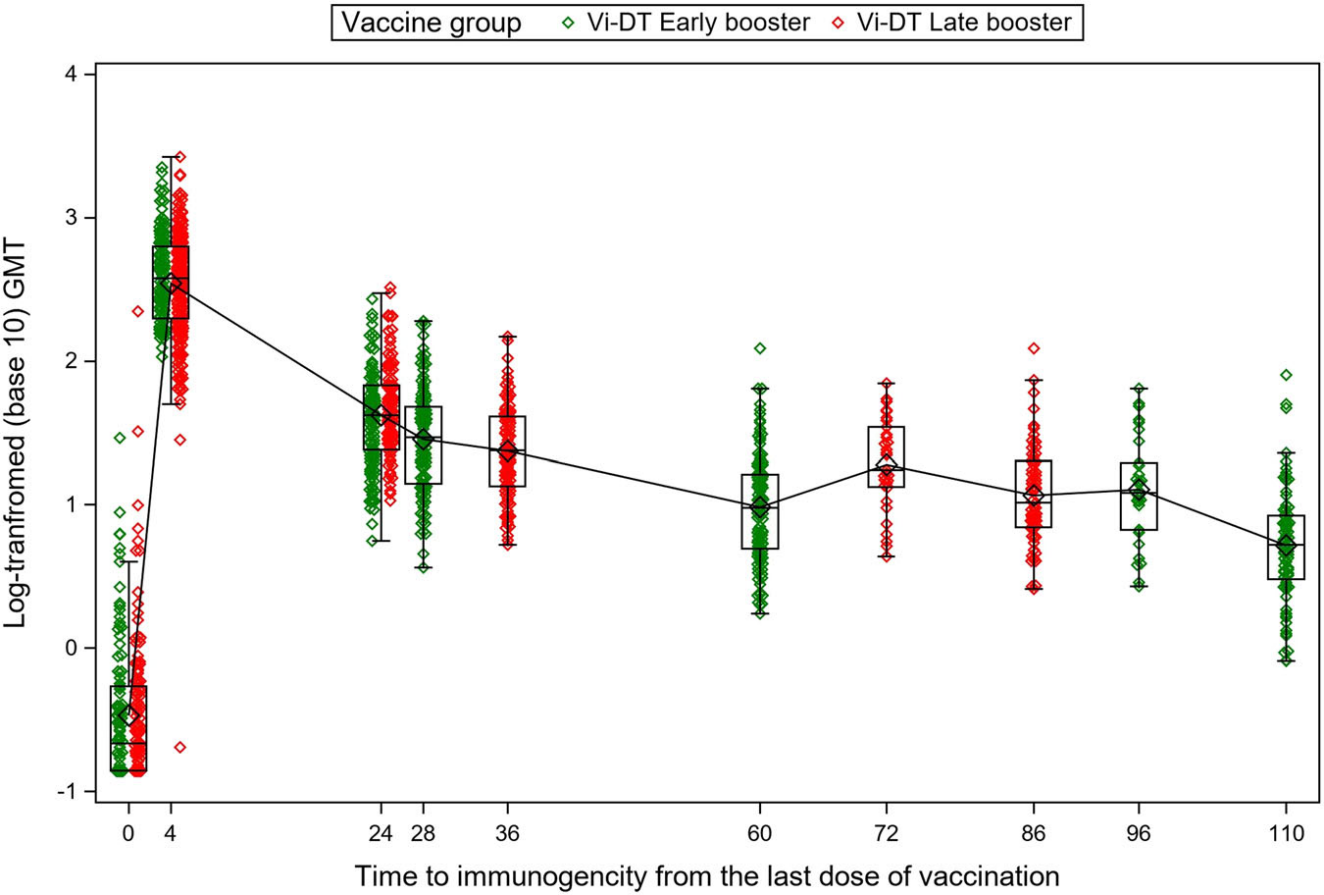
Geometric mean titers presented by time from the last dose of Vi-DT in late- and early booster groups receiving the Vi-DT vaccine among infants and toddlers. The *x* axis represents the duration between the last dose of vaccination and each immunogenicity timepoint, while the *y* axis represents the log_10_-transformed geometric mean titer (GMT) levels. For the late-booster group, the durations on the *x* axis represent the duration between the first dose and the immunogenicity timepoint; for the early booster group, the duration refers to the time between the first dose and immunogenicity timepoint for the 4-week and 24-week timepoints, while for 28, 60, 96, and 110, this represents the duration between the second dose and the immunogenicity timepoint.

To account for the differences in the duration between the last vaccination timepoint and immunogenicity blood draw, we computed a new variable representing the time-to-immunogenicity blood draw from the last dose of vaccination in weeks. We then plotted the GMTs in both groups against this time as shown in Fig. 4. It was evident that at comparable durations from the last-dose vaccination, there was no substantive difference in GMTs between the late- and early booster groups.

The seroconversion rates and GMTs in the per-protocol analysis set mirrored those in the immunogenicity-analysis set. Table 1 presents the seroconversion rates at weeks 60, 96, and 110 in late- and early booster Vi-DT groups.

**Table 1:** Seroconversion of anti-Vi IgG ELISA response—Immunogenicity analysis set.

Time point^a^	Late booster dose group		Early booster dose group		Comparator group		*P*-value^c^
	*N*	Seroconversion rate^b^ (95% CI)	*N*	Seroconversion rate^b^ (95% CI)	*N*	Seroconversion rate^b^ (95% CI)	
Day 0	114	–	114	–	57	–	–
Week 4	114	100.0 (96.74, 100.0)	114	100.0 (96.74, 100.0)	57	7.02 (2.76, 16.70)	
Week 24	112	99.11 (95.12, 99.84)	110	97.27 (92.29, 99.07)	55	21.82 (12.95, 34.37)	–
Week 28	112	99.11 (95.12, 99.84)	109	98.17 (93.56, 99.50)	55	21.82 (12.95, 34.37)	<0.0001 [1] 0.1458 [2] 0.5508 [3]
Week 60	111	95.50 (89.89, 98.06)	109	96.33 (90.94, 98.56)	N/A	N/A	0.7567 [5]
Week 96	29	89.66 (73.61, 96.42)	31	96.77 (83.81, 99.43)	N/A	N/A	0.3037 [8]
Week 110	76	88.16 (79.00, 93.64)	74	94.59 (86.91, 97.88)	N/A	N/A	0.1621 [9]
Week 114a	29	96.55 (82.82, 99.39)	N/A	N/A	N/A	N/A	–
Week 114b	76	100.0 (95.19, 100.0)	N/A	N/A	N/A	N/A	–
*P*-value within group^c^	0.6115 [6] 0.0030 [7]		0.5737 [4]		–		

*N/A* not applicable for the groups that completed the last visit.

^a^First Vi-DT dose at Day 0 in late booster and early booster groups. The early booster Vi-DT dose vaccination was provided at week 24 in early booster dose group. Late booster Vi-DT dose vaccination in late booster group was provided at either week 96 or week 110. Week 114a included subjects who received late boost dose at week 96; Week 114b included subjects who received the late boost dose at week 110.

^b^Proportion of participants who had 4-fold rise in titers compared to baseline (Day 0, Week 0).

^c^*P*-values from stratified Chi-square (Cochran-Mantel-Haenszel) test stratified by age. *P*-values within group from the stratified paired test were stratified by age.

[1] Secondary, Seroconversion of Anti-Vi IgG ELISA Response at Week 28 (early booster group vs. comparator).

Following comparison is tested without multiple testing adjustments, with no decision-making criteria.

[2] Seroconversion of Anti-Vi IgG ELISA Response at Week 4 vs. Week 28 (late booster vs. early booster).

[3] Seroconversion of Anti-Vi IgG ELISA Response at Week 28 (late booster vs. early booster).

[4] Seroconversion of Anti-Vi IgG ELISA Response at Week 24 vs. Week 28 of early booster dose.

[5] Seroconversion of Anti-Vi IgG ELISA Response at Week 60 (late booster vs. early booster).

[6] Seroconversion of Anti-Vi IgG ELISA Response at Week 96 vs. Week 114a of late booster group. *P*-value derived by Fisher’s exact test without age adjustment due to zero cells.

[7] Seroconversion of Anti-Vi IgG ELISA Response at Week 110 vs. Week 114b of late booster group. *P*-value derived by Fisher’s exact test without age adjustment due to zero cells.

[8] Seroconversion of Anti-Vi IgG ELISA Response at Week 96 (late booster vs. early booster).

[9] Seroconversion of Anti-Vi IgG ELISA Response at Week 110 (late booster vs. early booster).

### Safety and immunogenicity following booster vaccination in the late booster group

In a previous analysis, we reported that Vi-DT is safe and well-tolerated with comparable solicited and unsolicited adverse events within four weeks of the first dose and the early booster at 24 weeks compared with comparator groups^16^. Over 114 weeks of follow-up after the first dose, no safety signal was reported. A total of 21 serious adverse events (SAEs), none of which were assessed to be related to the investigational product, were reported. There was no death. Four local solicited adverse events related to the investigational product, five unlikely related, and one unrelated unsolicited adverse event was reported within four weeks following the booster dosing in the late-booster group. All were assessed as mild to moderate in severity.

### Seroconversion and GMT following booster vaccination in the late booster group

We then assessed the immune response in the late-booster Vi-DT group following booster vaccination at two timepoints—96 and 110 weeks using the immunogenicity-analysis set. Participants who received the booster at week 96 (*n* = 29) underwent immunogenicity blood draw at week 114 where the seroconversion rate was 96.55% (95% CI: 82.82, 99.39), which was not a statistically significant increase from the prebooster seroconversion rate (*p* = 0.612). Whereas, in participants who received the booster dose at week 110 and immunogenicity blood draw at week 114, seroconversion rate increased to 100.0% (95% CI: 95.19, 100.0, *p* = 0.003). A significant increase from the prebooster values was observed in the GMTs at week 114 following booster dosing at both time points—43.94 (95% CI: 17.97, 107.47) 18 weeks following booster dose at week 96 (*p* < 0.0001) and 351.76 (95% CI: 265.01, 466.93) four weeks following booster dose at week 110 (*p* < 0.0001). Notably, the GMT at 4 weeks post booster dose is comparable to the level of primary response following the first dose of Vi-DT.

The per-protocol analysis mirrored seroconversion and GMT values following booster dosing. GMT and seroconversion values following booster vaccination are presented in Table 1 and Table 2, respectively.

**Table 2:** Geometric mean titers (GMTs) of Anti-Vi IgG ELISA response—immunogenicity set.

Timepoint^a^	Late booster group			Early booster group			Comparator group			*P*-value^b^
	N	GMT (95% CI)^b^	GSD^b^	N	GMT (95% CI)^b^	GSD^b^	N	GMT (95% CI)^b^	GSD^b^	
Day 0	114	0.32 (0.26, 0.41)	3.08	114	0.38 (0.30, 0.47)	3.59	57	0.43 (0.31, 0.59)	4.12	–
Week 4	114	420.03 (364.16,484.49)	2.00	114	470.14 (407.60,542.29)	1.96	57	0.41 (0.34, 0.50)	3.20	–
Week 24	112	36.82 (31.47, 43.08)	2.23	110	46.99 (40.10, 55.06)	2.06	55	0.53 (0.42, 0.66)	3.55	–
Week 28	112	28.70 (23.37, 35.25)	2.36	109	201.28 (163.41, 247.92)	2.83	55	0.61 (0.46, 0.82)	4.94	<0.0001 [1]
Week 60	111	9.60 (8.34, 11.05)	2.38	109	23.01 (19.96, 26.52)	2.07	N/A	N/A	N/A	<0.0001 [4]
Week 96	29	7.13 (3.60, 14.14)	2.39	31	11.18 (5.97, 20.90)	2.09	N/A	N/A	N/A	0.0343 [6]
Week 110	76	5.50 (4.44, 6.80)	2.52	74	11.95 (9.65, 14.81)	2.19	N/A	N/A	N/A	<0.0001 [8]
Week 114a	29	43.94 (17.97, 107.47)	2.52	N/A	N/A	N/A	N/A	N/A	N/A	–
Week 114b	76	351.76 (265.01, 466.93)	2.77	N/A	N/A	N/A	N/A	N/A	N/A	–
*P*-value^c^ within group		<0.0001 [5] <0.0001 [7] 0.3188 [9]			<0.0001 [2] <0.0001 [3]			–		0.0005 [10]

*N/A* not applicable for the groups that completed the last visit, *LOD* (Limit of Detection) 0.14 for ELISA.

^a^First Vi-DT dose at Day 0 in late booster and early booster groups. The early booster Vi-DT vaccination at week 24 in early booster group. Late booster Vi-DT dose vaccination in the late booster at either Week 96 or Week 110. Week 114a includes subjects who received post boost dose at Week 96; Week 114b includes subjects who received post boost dose at Week 110.

^b^Geometric Mean Titers (unit: IU/ml) and Geometric Standard Deviation. Geometric Mean Fold rise from baseline (Day 0) to post dose.

^c^*P*-values for comparison of GMTs and fold difference within the group have been derived using analysis of variance adjusting for age strata.

[1] Secondary, GMT of Anti-Vi IgG ELISA Response at Week 28 (late booster vs. early booster.

Following comparison is tested without multiple testing adjustment, no decision-making criteria

[2] GMT of Anti-Vi IgG ELISA Response at Week 4 vs. Week 28 of early booster.

[3] GMT of Anti-Vi IgG ELISA Response at Week 24 vs. Week 28 of early booster.

[4] GMT of Anti-Vi IgG ELISA Response at Week 60 (late booster vs. early booster).

[5] GMT of Anti-Vi IgG ELISA Response at Week 96 vs. Week 114 of late booster group. Included subjects who received boost dose at Week 96.

[6] GMT of Anti-Vi IgG ELISA Response at Week 96 (late booster vs. early booster).

[7] GMT of Anti-Vi IgG ELISA Response at Week 110 vs. Week 114 of late booster group. Included subjects who received boost dose at Week 110.

[8] GMT of Anti-Vi IgG ELISA Response at Week 110 (late booster vs. early booster).

[9] GMT of Anti-Vi IgG ELISA Response at Week 4 vs. Week 114 of late booster group. Excluded subject who received late boost dose at Week 96.

## Discussion

In the presented analysis, we demonstrated that Vi-DT is safe up to 114 weeks after the first dose with a satisfactory safety profile 4 weeks after receiving the late-booster dose administered at 110 weeks. Furthermore, we showed that the prebooster serological response following the first dose in the late-booster group and the post-booster values in the early booster Vi-DT groups in infants and toddlers persisted at week 110. The post-booster GMTs in the early booster group were however significantly higher than the prebooster values in the late-booster group at each follow-up timepoint. The late-booster Vi-DT dose at 110 weeks elicited a significant increase in seroconversion and GMTs at four weeks receiving the booster dose, and the GMT levels at 4 weeks post booster dose were comparable to the primary response following the first dose of Vi-DT^15,16^. Notably, this immune response appeared more robust than the response observed following early boosting at 24 weeks, which implies that a late-booster dose of Vi-DT 27.5 months after administration of the first dose may be a better vaccination strategy albeit the lack of well-defined protective thresholds.

Our results need to be interpreted with caution considering some relevant limitations. First, due to the COVID-19 pandemic in the Philippines, all participants could not receive the late-booster dose at a similar timepoint and could not be pooled for comparing immunogenicity in the late-booster and early booster Vi-DT groups at week 110. Second, the late-booster and early booster groups did not provide blood samples at comparable timepoints after each subject received his/her last dose of Vi-DT, which denotes the time after first-dose vaccination for the late-booster group and after week 24 for the early booster group. Therefore, the comparison at each timepoint might have been influenced by the differences in the window of time in which each participant received their last dose of vaccination, and when their blood sample was taken for immunogenicity tests. This difference might explain the significantly higher GMTs observed in the post-booster GMTs in the early booster group at the 60-, 96-, and 110-week immunogenicity assessments. This argument was further supported by the assessment of immune responses at comparable window of time from the last-dose of vaccination in the late- and early booster groups, which demonstrated comparable GMTs at approximately similar windows from the last dose vaccination. Third, there was a significant number of children whose screen failed due to mild laboratory abnormalities, including anemia and out-of-range liver enzymes at baseline, which is a common scenario in the study setting. Finally, the lack of clearly defined immunogenicity correlates of protection against typhoid fever restricts the interpretation of immune persistence in the context of prolonged vaccine protection against infection and/or disease. A recent analysis of human-challenge models for correlates of protection showed that the serum bactericidal antibody (SBA) levels do not correlate with clinical disease and that these values poorly correlate with anti-Vi IgG and IgM titers^19^. Similarly for Vi IgG and IgA, there was no significant correlation between fold change and development of typhoid fever even though a nonsignificant positive association was observed between fold increases in Vi IgG and development of typhoid fever^20^.

Despite these limitations, our study has several strengths that imparted internal and external validity to the results. The study presents the first investigation of long-term immune persistence following vaccination with Vi-DT in infants and toddlers, a vaccine that has shown robust immune response in short-to-midterm evaluations^14–16^. Strict adherence to standards of good clinical and laboratory practice, quality control, and assurance methods warranted assessment of participant safety, including long-term safety profile, and enabled the tests to generate high-quality data. Laboratory-analysis methods for immunogenicity were performed using quality-assured internationally accredited assays to enable comparability and validity of the study results.

Our results lend optimism to the notion that typhoid-conjugate vaccines potentially elicit long-term immune responses in children under 2 years of age more so than Vi-polysaccharide vaccines, owing to the T-cell-mediated immune memory following vaccination with protein-conjugated vaccines^21,22^. The results also indicate that, while immune responses following the first dose of Vi-DT persisted for as long as 110 weeks of follow-up, a late booster at 110 weeks did not boost the response more than that observed following vaccination with the first dose of Vi-DT^14–16^. This finding implies that a booster dose every 2–3 years after administration of the first dose might confer better protection against typhoid fever in children, given the lower GMTs at the 24- and 28-month timepoint, a trend similarly demonstrated in trials involving other typhoid-conjugate vaccines^18^.

Extending the follow-up period for immune persistence, and evaluation of immune-response trends against clinical efficacy in protecting from typhoid fever in long-term follow-up studies would provide a more holistic understanding of the need for and timing of a booster dose with Vi-DT. Ongoing cluster-randomized trials evaluating the real-world effectiveness of Typbar TCV (Vi-TT) coupled with long-term immunogenicity assessments will provide critical evidence toward immune persistence and vaccine efficacy and effectiveness^23,24^. We recommend similar investigations of long-term immunogenicity and efficacy/effectiveness evaluations for Vi-DT.

## Methods

The detailed trial design and methods have been described elsewhere^15,16^.

### Trial design

Randomized, controlled, observer-blinded phase-2 study (Clinicaltrials.gov: NCT03527355). Infants and toddlers 6–23 months of age were randomized in a 2:2:1 ratio to one of three groups: (1) group which received a first dose Vi-DT vaccine followed by “early booster” at 24 weeks, (2) the group that received a first-dose of Vi-DT followed by “late booster” at 96 or 110 weeks, and (3) a comparator group. Randomization to one of the treatment groups was done considering the proportional assignment of the three age strata—6–9 months, 9–12 months, and 13–23 months, with 95 children assigned to each age stratum.

### Participants

Detailed inclusion/exclusion criteria have been recently published^16^. Briefly, participants were randomized to early booster Vi-DT (*n* = 114), late-booster Vi-DT (*n* = 114), and comparator (*n* = 57) groups at the Research Institute for Tropical Medicine (RITM), Manila, Republic of the Philippines. Recruitment occurred at the RITM or nearby health facilities where parents and legally acceptable representatives (LAR) visiting these facilities between April and July 2018 for regular immunizations or medical checkup of their children were invited to participate in the study. Measles, mumps, and rubella (MMR) vaccine was provided to children 9–12 months of age—where co-administration with Vi-DT was done in 76 children. As reported previously, there is no evidence of immune interference between MMR and Vi-DT^16^.

### Vaccines and vaccination schedule

Table 3 presents the vaccination schedule and study procedures.

**Table 3:** Vaccination schedule and outcome assessment in the two Vi-DT groups and the comparator.

Timepoint	Late booster Vi-DT group	Early booster Vi-DT group	Comparator group
Baseline	First dose of Vi-DT immunogenicity blood draw	First dose of Vi-DT immunogenicity blood draw	Normal saline. TRIMOVAX^®^ for 9–12 months
1 week	Solicited AEs		
4 weeks	Unsolicited AEs and Immunogenicity blood draw		
24 weeks	FluQuadri^TM^ Immunogenicity Blood draw	Early booster of Vi-DT Immunogenicity Blood draw	FluQuadri^TM,^ TRIMOVAX^®^ for 9–12 months
25 weeks	Solicited AEs		
28 weeks	Unsolicited AEs and Immunogenicity blood draw		
60 weeks	Immunogenicity Blood draw		
96 weeks	Booster dose of Vi-DT* Immunogenicity blood draw*	Immunogenicity Blood draw**	
97 weeks	Solicited AEs		
110 weeks	Late booster dose of Vi-DT* Immunogenicity Blood draw**	Immunogenicity Blood draw**	
111 weeks	Solicited AEs		
114 weeks	Unsolicited AEs and Immunogenicity Blood draw**		

*29 children received the late booster dose at week 96 as planned in the original proposal; 76 children received the late booster dose at week 110.

**Immunogenicity blood draw was performed for both groups at a similar timepoint (week 114)—4 and 18 weeks after the late booster dosing.

In the intervention arm, the early booster group received two doses of Vi-DT with a 24-week window with the first dose administered at enrollment. The late-booster group received the first dose of Vi-DT at enrollment and the booster dose at 96 or 110 weeks after. The Vi-DT vaccine contains 25 μg of purified Vi polysaccharide (*S*. Typhi C6524) and 37 μg of diphtheria toxoid (*Corynebacterium diphtheriae* PW No. 8), respectively. The Vi-DT vaccine was manufactured, packaged, and labeled by SK Bioscience, Republic of Korea. Storage was at 2–8 °C as recommended by the manufacturer.

Participants in the comparator arm received one of three interventions. First, a placebo (0.5 mL of 0.9% sodium chloride packaged as 2- or 5-mL colorless ampoules) was administered to the comparator group at the time when the other two groups received their first dose of Vi-DT vaccines. Second, the comparator group and the late-booster Vi-DT group received 0.25 mL of FluQuadri^TM^ of inactivated quadrivalent influenza vaccine (Sanofi Pasteur, France) at the time when the early booster Vi-DT group received their second dose of Vi-DT. Third, all children at 9–12 months of age, regardless of their group association, received 0.5 mL of TRIMOVAX^®^, attenuated measles, mumps, and rubella vaccine (Sanofi Pasteur, France) at any follow-up as required by national immunization programs.

### Study procedures and follow-up

Details of randomization and blinding were published previously^16^. In brief, children were randomized by age strata—6–9 months, 9–12 months, and 12–23 months of age. Immediate reactogenicity, solicited, and unsolicited adverse events were assessed within the first four weeks of each vaccination. A very good safety profile of Vi-DT was shown in our recent publications^14–16^. Safety assessment and coding were done using MedDRA (version 21.0)^25^. Blood samples were collected before vaccination and 4, 24, 28, 60, 96, 110, and 114 weeks post first dose for immunogenicity assessment. Two interim analyses were performed at weeks 4 and 28 post first dose of Vi-DT^16,26^.

Herein, we present a follow-up analysis of the immune persistence at 60, 96, and 110 weeks in the late- and early booster Vi-DT groups, and the immune response at 4 and 18 weeks after receiving a booster dose of Vi-DT to the late-booster group, which was at weeks 96 and 110 since this group received the first dose of Vi-DT. Due to the COVID-19 pandemic, the booster dose to the late-booster Vi-DT group was provided at two different points in time—at weeks 96 (*n* = 29) and 110 (*n* = 76) instead of the initial plan to unilaterally administer the booster dose at week 96 post first dose of Vi-DT. Furthermore, the post-booster immunogenicity blood draw for those who received Vi-DT booster dose at week 96 was collected at week 114 (18 weeks since the subject received his/her booster dose). For participants who received the booster dose at week 110, a blood draw for immunogenicity was performed at week 114 (4 weeks post booster dose). Unblinding of the study team was done at week 36 post first dose.

### Outcome assessment

Safety and immunogenicity assessments within 4 weeks of the first dose and the early booster of Vi-DT were described in recent publications^14–16^. Seroconversion rates and geometric mean titers (GMTs) of anti-Vi IgG were further determined at weeks 60, 96, 110, and 114 post first dose of Vi-DT in both the early and late-booster Vi-DT groups. In this analysis, safety and reactogenicity within 4 weeks of booster dose and serious adverse events (SAE) during the total period of follow-up have been presented.

Immunogenicity following vaccination with Vi-DT was assessed using anti-Vi IgG ELISA as previously described^27^. Antibody titers (international unit, IU/ml) were determined based on the international standard serum (NIBSC 16/138). Seroconversion was defined as a fourfold rise from baseline in the anti-Vi-IgG titers.

### Sample size

For this follow-up analysis of the phase-2 randomized-controlled trial^16^, a minimum sample size of 70 participants in each Vi-DT group at the respective follow-up time points provided >80% power to assess noninferiority of the GMTs in the late-booster compared with the early booster Vi-DT groups, using the one-sided test at a 0.025 significance level (85% for a significance level of 0.0125) and assuming a true GMT ratio of 1, antibody-titer coefficient of variation of 3.0, and a noninferiority margin of the ratio of 0.5 (WHO Technical Report Series 924). The seroconversion rate and coefficient of variation of GMT were assumed conservatively based on the phase-1 data^14^. For the late-booster dose evaluation, 76 participants from late-booster Vi-DT group who received the booster dose at week 110 provided 80% power to assess the difference in seroconversion rates between the pre- and post-booster timepoints.

### Randomization and masking

As described previously^16^, randomization codes were generated by an independent biostatistician. Block randomization to balance groups by age strata and observer blinding were employed until 36 weeks of follow-up. Since Vi-DT and the comparator vaccines had differing visual presentations, double-blinding was not possible. After 36 weeks, the placebo group was terminated from the study while the early and late-booster Vi-DT groups continued follow-up as an open-label study.

### Statistical analysis

The intention-to-treat (ITT) analysis included all participants randomized in the study. The safety-analysis set was a subset of ITT analysis among those who received at least one dose of the investigational vaccine. The immunogenicity-analysis set was also a subset of the ITT analysis and included those who received at least one dose of investigational vaccines and had at least one post-baseline immunogenicity assessment.

The per-protocol (PP) analysis set included subjects who did not have major protocol violations defined as those compromising the scientific integrity of the study. Analysis of covariance was used to adjust for baseline titers, stratification, and imbalances in baseline characteristics as applicable. Imputation was not done for missing immunogenicity data.

The proportion of participants with at least a 4-fold rise of anti-Vi IgG antibody titer compared with baseline was assessed using the Cochran–Mantel–Haenszel (CMH) test. The GMT was calculated by multiplying all values and taking the *n*^*th*^ root of the average, where n is the number of subjects with available data^28,29^. Reverse cumulative distribution curves (RCDC) comparing the distribution of GMTs between the early and late-booster Vi-DT groups were generated for each immunogenicity blood-draw timepoint.

### Ethical considerations

The study was approved by the ethical review committees of the RITM and the International Vaccine Institute (IVI). Regulatory approval was secured from the Philippines Food and Drug Administration (PFDA). The study was conducted following the principles of ICH-GCP E6 (R2), the Declaration of Helsinki, Council for International Organizations of Medical Science (CIOMS), and applicable local and regional ethics and regulatory requirements. Participation was completely voluntary, and all parents or legally acceptable representatives (LAR) signed informed consent. A copy of the informed-consent document was provided to the parent or legally acceptable representative. Confidentiality of all participants was maintained throughout the study.

### Reporting summary

Further information on research design is available in the Nature Research Reporting Summary linked to this article.

## Supplementary information

REPORTING SUMMARY

## Data Availability

The datasets generated during and/or analyzed during the current study are available from the corresponding author on reasonable request.

## Code availability

No computer code or mathematical algorithm was used in this study.

## References

[1] Brockett S (2020). Associations among water, sanitation, and hygiene, and food exposures and typhoid fever in case? Control studies: a systematic review and meta-analysis. Am. J. Trop. Med. Hyg..

[2] GBD 2017 Typhoid and Paratyphoid Collaborators. The global burden of typhoid and paratyphoid fevers: a systematic analysis for the Global Burden of Disease Study 2017. *Lancet Infect. Dis*. **19**, 369–381 (2019). 10.1016/S1473-3099(18)30685-6 PMC6437314 30792131

[3] Marks F (2017). Incidence of invasive salmonella disease in sub-Saharan Africa: a multicentre population-based surveillance study. Lancet Glob. Health.

[4] Park SE (2018). The phylogeography and incidence of multi-drug resistant typhoid fever in sub-Saharan Africa. Nat. Commun..

[5] Rasheed MK, Hasan SS, Babar ZU, Ahmed SI (2019). Extensively drug-resistant typhoid fever in Pakistan. Lancet Infect. Dis..

[6] Qureshi S (2020). Response of extensively drug resistant Salmonella Typhi to treatment with meropenem and azithromycin, in Pakistan. PLoS Negl. Trop. Dis..

[7] Cadoz M (1998). Potential and limitations of polysaccharide vaccines in infancy. Vaccine.

[8] Rijkers GT, Sanders EAM, Breukels MA, Zegers BJM (1996). Responsiveness of infants to capsular polysaccharides: implications for vaccine development. Rev. Med. Microbiol..

[9] World Health Organization. (2019). Typhoid vaccines: WHO position paper, March 2018 - Recommendations. Vaccine.

[10] Syed KA (2020). Review on the recent advances on typhoid vaccine development and challenges ahead. Clin. Infect. Dis..

[11] Qadri F (2021). Protection by vaccination of children against typhoid fever with a Vi-tetanus toxoid conjugate vaccine in urban Bangladesh: a cluster-randomised trial. Lancet.

[12] Patel PD (2021). Safety and efficacy of a typhoid conjugate vaccine in Malawian children. N. Engl. J. Med..

[13] Shakya M (2019). Phase 3 efficacy analysis of a typhoid conjugate vaccine trial in Nepal. N. Engl. J. Med..

[14] Capeding M (2018). Safety and immunogenicity of a Vi-DT typhoid conjugate vaccine: phase I trial in healthy Filipino adults and children. Vaccine.

[15] Capeding MR (2020). Immunogenicity, safety and reactogenicity of a Phase II trial of Vi-DT typhoid conjugate vaccine in healthy Filipino infants and toddlers: a preliminary report. Vaccine.

[16] Capeding, M. R. et al. Safety and immunogenicity of Vi-DT conjugate vaccine among 6-23-month-old children: Phase II, randomized, dose-scheduling, observer-blind Study. *EClinicalMedicine*, 10.1016/j.eclinm.2020.100540 (2020). 10.1016/j.eclinm.2020.100540 PMC7599314 33150320

[17] Cui C (2010). Physical and chemical characterization and immunologic properties of Salmonella enterica serovar typhi capsular polysaccharide-diphtheria toxoid conjugates. Clin. Vaccin. Immunol..

[18] Mohan VK (2015). Safety and immunogenicity of a Vi polysaccharide-tetanus toxoid conjugate vaccine (Typbar-TCV) in healthy infants, children, and adults in typhoid endemic areas: a multicenter, 2-cohort, open-label, double-blind, randomized controlled phase 3 study. Clin. Infect. Dis..

[19] Jones, E. et al. A Salmonella Typhi controlled human infection study for assessing correlation between Bactericidal antibodies and protection against infection induced by typhoid vaccination. *Microorganisms*, 10.3390/microorganisms9071394 (2021). 10.3390/microorganisms9071394 PMC8304662 34203328

[20] Jin, C. et al. Vi-specific serological correlates of protection for typhoid fever. *J. Exp. Med.*, 10.1084/jem.20201116 (2020). 10.1084/jem.20201116 PMC7668386 33180929

[21] McCool TL, Harding CV, Greenspan NS, Schreiber JR (1999). B- and T-cell immune responses to pneumococcal conjugate vaccines: divergence between carrier- and polysaccharide-specific immunogenicity. Infect. Immun..

[22] Sallusto F, Lanzavecchia A, Araki K, Ahmed R (2010). From vaccines to memory and back. Immunity.

[23] Haselbeck, A. H. et al. Evaluation of typhoid conjugate vaccine effectiveness in Ghana (TyVEGHA) using a cluster-randomized controlled phase IV trial: trial design and population baseline characteristics. *Vaccines*, 10.3390/vaccines9030281 (2021). 10.3390/vaccines9030281 PMC8003794 33808924

[24] Theiss-Nyland K (2019). Assessing the impact of a Vi-polysaccharide conjugate vaccine in preventing typhoid infection among bangladeshi children: a protocol for a phase IIIb trial. Clin. Infect. Dis.: Off. Publ. Infect. Dis. Soc. Am..

[25] Medical Dictionary for Regulatory Activities – MedDRA. https://www.meddra.org/ (2019). 10.2165/00002018-199920020-00002 10082069

[26] Capeding, M. R. et al. Immunogenicity, safety and reactogenicity of a Phase II trial of Vi-DT typhoid conjugate vaccine in healthy Filipino infants and toddlers: a preliminary report. *Vaccine*, 10.1016/j.vaccine.2019.09.074 (2019). 10.1016/j.vaccine.2019.09.074 PMC7273193 31585725

[27] Rijpkema S (2018). Establishment of the first International Standard for human anti-typhoid capsular Vi polysaccharide IgG. Biologicals.

[28] Sahastrabuddhe S, Saluja T (2019). Overview of the typhoid conjugate vaccine pipeline: current status and future plans. Clin. Infect. Dis..

[29] Lee JS, Mogasale VV, Mogasale V, Lee K (2016). Geographical distribution of typhoid risk factors in low and middle income countries. BMC Infect. Dis..

