# Supplementary material for: Immune persistence and response to booster dose of Vi-DT vaccine at 27.5 months post-first dose
Source: NPJ Vaccines. 2022 Jan 27;7:12. doi: 10.1038/s41541-022-00434-8 (PMC8795159; doi:10.1038/s41541-022-00434-8)
Supplement: Supplementary file 1 — REPORTING SUMMARY [file 41541_2022_434_MOESM1_ESM.pdf]

nature portfolio

Corresponding author(s): Dr Birkneh Tilahun Tadesse
MS ID: NPJVACCINES-01628

Last updated by author(s): Oct 27, 2021

Reporting Summary

Nature Portfolio wishes to improve the reproducibility of the work that we publish. This form provides structure for consistency and transparency in reporting. For further information on Nature Portfolio policies, see our Editorial Policies and the Editorial Policy Checklist.

Statistics

For all statistical analyses, confirm that the following items are present in the figure legend, table legend, main text, or Methods section.

n/a	Confirmed	
☐	☒	The exact sample size (n) for each experimental group/condition, given as a discrete number and unit of measurement
☐	☒	A statement on whether measurements were taken from distinct samples or whether the same sample was measured repeatedly
☐	☒	The statistical test(s) used AND whether they are one- or two-sided<br>*Only common tests should be described solely by name; describe more complex techniques in the Methods section.*
☐	☒	A description of all covariates tested
☐	☒	A description of any assumptions or corrections, such as tests of normality and adjustment for multiple comparisons
☐	☒	A full description of the statistical parameters including central tendency (e.g. means) or other basic estimates (e.g. regression coefficient) AND variation (e.g. standard deviation) or associated estimates of uncertainty (e.g. confidence intervals)
☐	☒	For null hypothesis testing, the test statistic (e.g. F, t, r) with confidence intervals, effect sizes, degrees of freedom and P value noted<br>*Give P values as exact values whenever suitable.*
☒	☐	For Bayesian analysis, information on the choice of priors and Markov chain Monte Carlo settings
☒	☐	For hierarchical and complex designs, identification of the appropriate level for tests and full reporting of outcomes
☒	☐	Estimates of effect sizes (e.g. Cohen's d, Pearson's r), indicating how they were calculated

Our web collection on statistics for biologists contains articles on many of the points above.

Software and code

Policy information about availability of computer code

Data collection	Data were collected in an electronic data capture system (EDC), which is validated.
Data analysis	SAS software was used for the data analysis.

For manuscripts utilizing custom algorithms or software that are central to the research but not yet described in published literature, software must be made available to editors and reviewers. We strongly encourage code deposition in a community repository (e.g. GitHub). See the Nature Portfolio guidelines for submitting code & software for further information.

Data

Policy information about availability of data

All manuscripts must include a data availability statement. This statement should provide the following information, where applicable:

- Accession codes, unique identifiers, or web links for publicly available datasets
- A description of any restrictions on data availability
- For clinical datasets or third party data, please ensure that the statement adheres to our policy

The datasets generated during and/or analyzed during the current study are available from the corresponding author on reasonable request.

Field-specific reporting

Please select the one below that is the best fit for your research. If you are not sure, read the appropriate sections before making your selection.

☒ Life sciences ☐ Behavioural & social sciences ☐ Ecological, evolutionary & environmental sciences

For a reference copy of the document with all sections, see nature.com/documents/nr-reporting-summary-flat.pdf

Life sciences study design

All studies must disclose on these points even when the disclosure is negative.

Sample size	285
Data exclusions	Two participants did not receive the second dose of Vi-DT while five participants – two each in the late booster and early booster groups, and one participant in the comparator group – had delayed 6-month vaccination. An additional participant each from the late booster and early booster Vi-DT groups was excluded at the time point when each received their booster shot, due to the administrators missing their blood sample to conduct an immunogenicity test.
Replication	Quality assurance procedures and audit were conducted to ensure reproducibility.
Randomization	Block randomization to balance groups by age strata and observer blinding were employed until 36 weeks of follow-up. Since Vi-DT and the comparator vaccines had differing visual presentations, double-blinding was not possible.
Blinding	The trial was double blind.

Reporting for specific materials, systems and methods

We require information from authors about some types of materials, experimental systems and methods used in many studies. Here, indicate whether each material, system or method listed is relevant to your study. If you are not sure if a list item applies to your research, read the appropriate section before selecting a response.

Materials & experimental systems

n/a	Involved in the study	
☐	☒	Antibodies
☒	☐	Eukaryotic cell lines
☒	☐	Palaeontology and archaeology
☒	☐	Animals and other organisms
☐	☒	Human research participants
☐	☒	Clinical data
☒	☐	Dual use research of concern

Methods

n/a	Involved in the study	
☒	☐	ChIP-seq
☒	☐	Flow cytometry
☒	☐	MRI-based neuroimaging

Antibodies

Antibodies used	Immunogenicity analysis as anti Vi IgG antibody titers were measured.
Validation	Good manufacturing practices were followed and tests were performed in quality assured labs.

Human research participants

Policy information about studies involving human research participants

Population characteristics	Children 6 months to 23 months
Recruitment	A total of 285 children were enrolled from 515 screened participants. Detailed inclusion/exclusion criteria have been recently published. Briefly, participants were randomized to early booster Vi-DT (n=114), late booster Vi-DT (n=114), and comparator (n=57) groups at the Research Institute for Tropical Medicine (RITM), Manila, Republic of the Philippines. Recruitment occurred at the RITM or nearby health facilities where parents and legally acceptable representatives (LAR) visiting these facilities between April and July 2018 for regular immunizations or medical check-up of their children were invited to participate in the study. Measles, mumps, and rubella (MMR) vaccine was provided to children 9 to 12 months of age – where co-administration with Vi-DT was done in 76 children. As reported previously, there is no evidence of immune interference between MMR and Vi-DT.

Ethics oversight	International Vaccine Institute (IVI) IRB and Philippines FDA

Note that full information on the approval of the study protocol must also be provided in the manuscript.

Clinical data

Policy information about clinical studies

All manuscripts should comply with the ICMJE guidelines for publication of clinical research and a completed CONSORT checklist must be included with all submissions.

Clinical trial registration	NCT03527355
Study protocol	A Phase II, Randomized, Dose-scheduling, Observer-Blinded Study to Assess the Safety, Reactogenicity and Immunogenicity of Vi-DT Conjugate Vaccine in 6-23-Month old Healthy Filipino Infants and Toddlers
Data collection	Collected using eCRF in EDC.
Outcomes	Safety and Immunogenicity of early and late booster Vi-DT.
